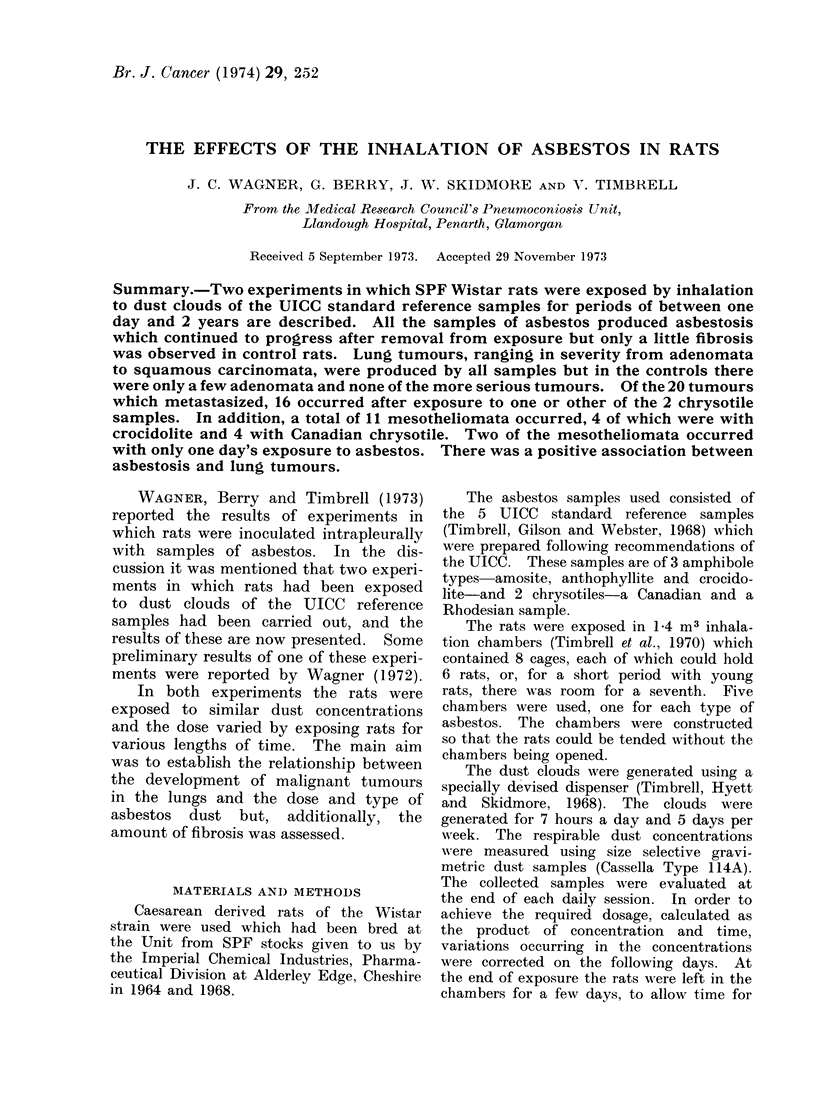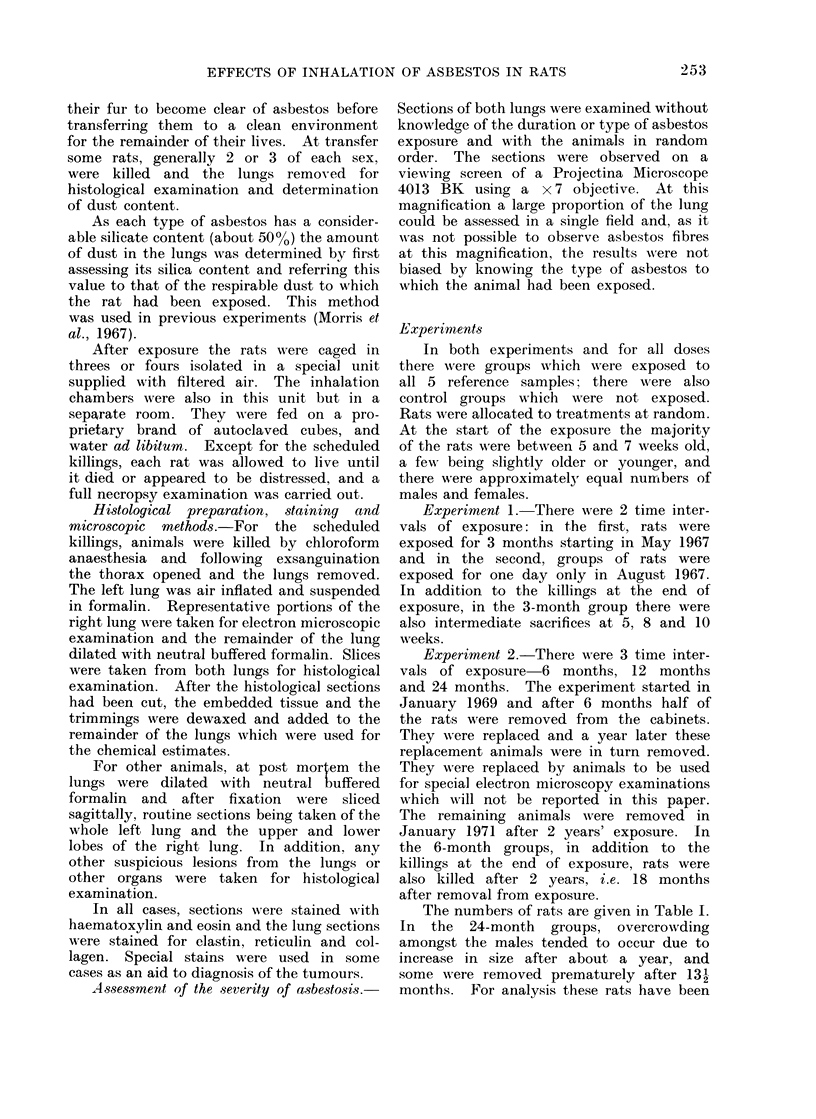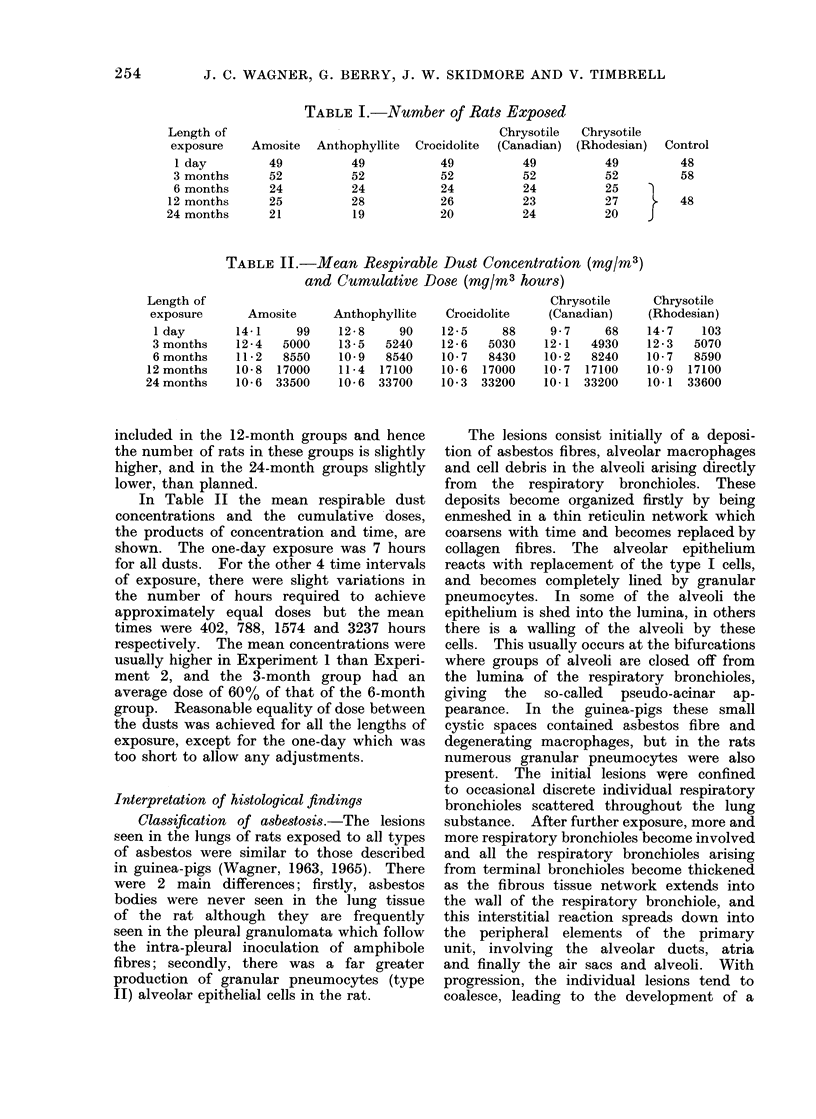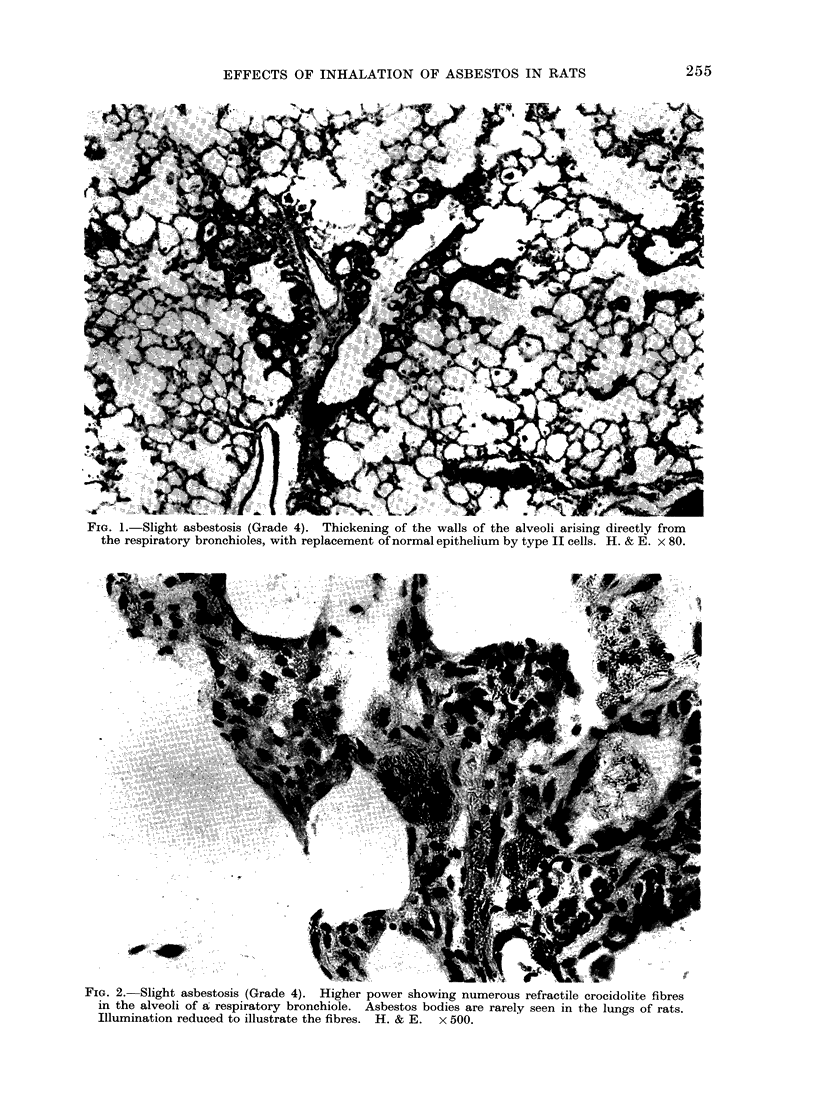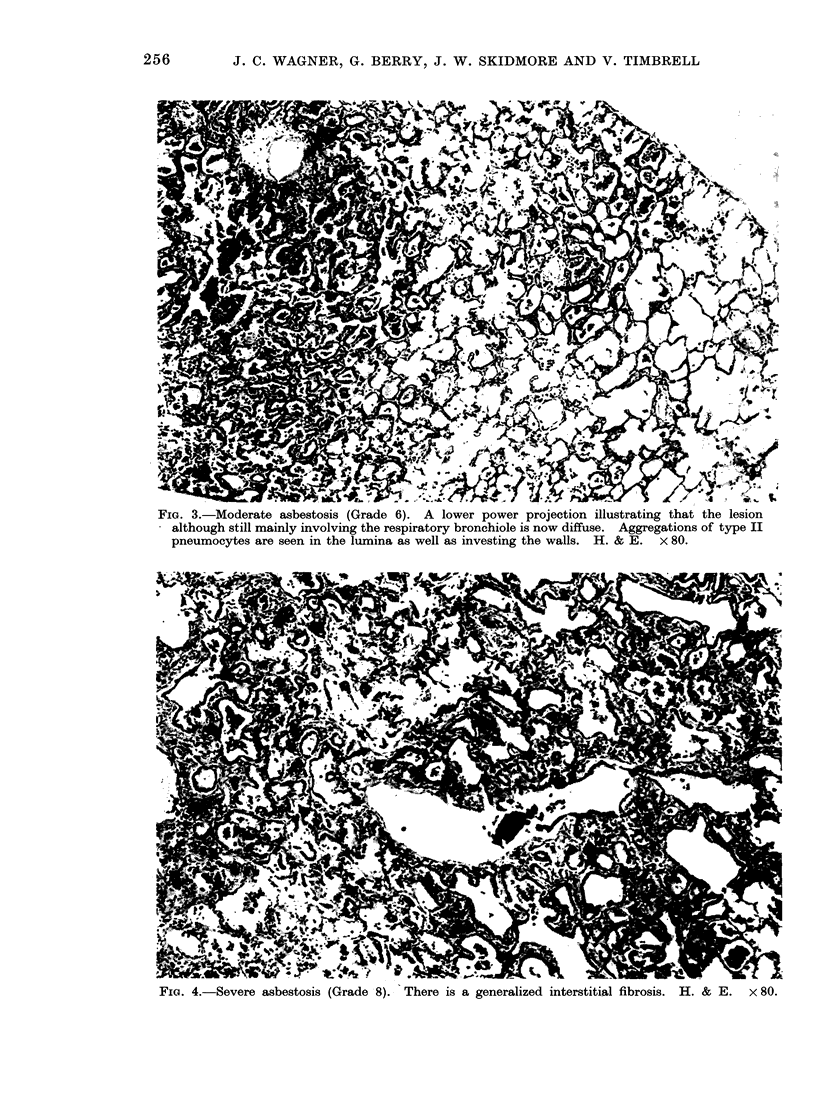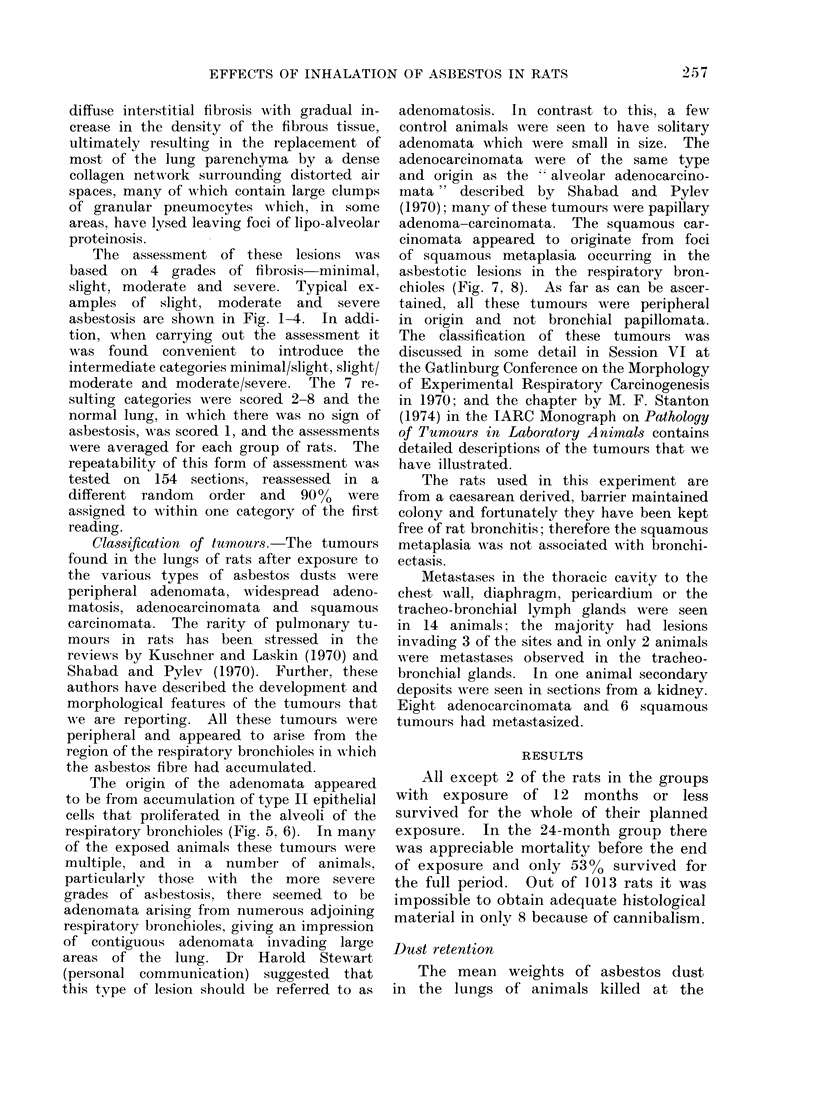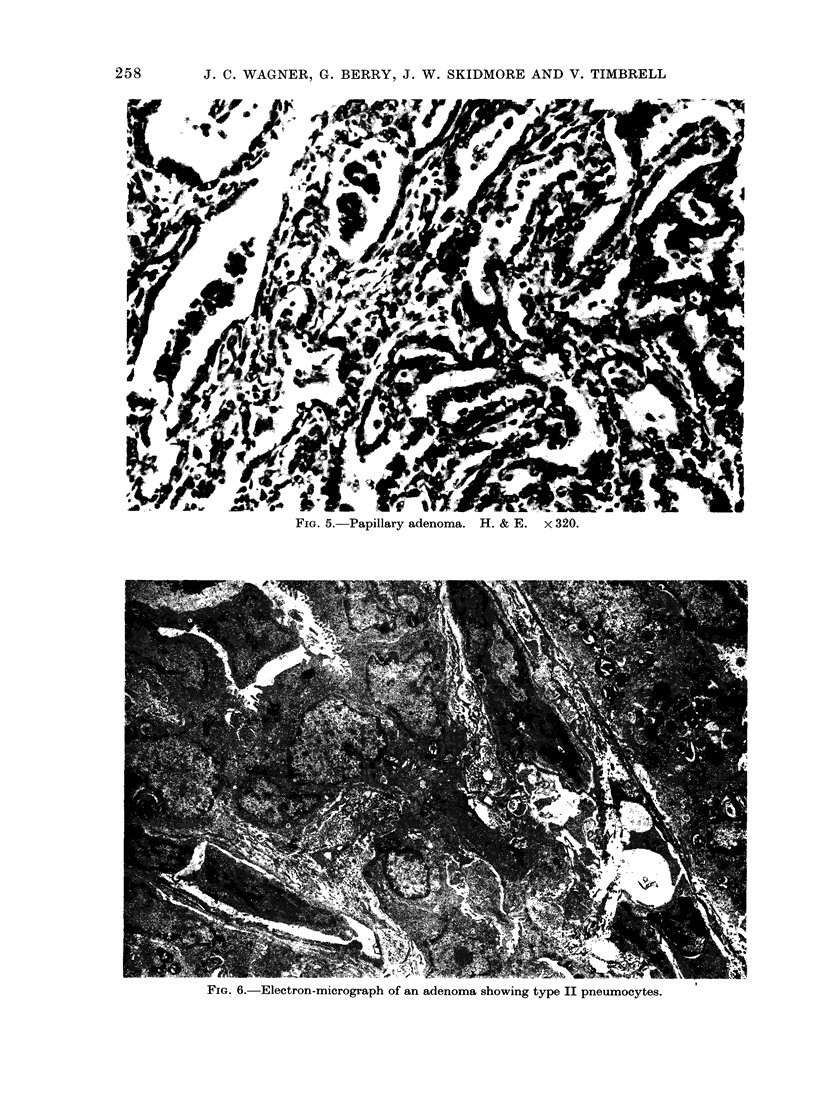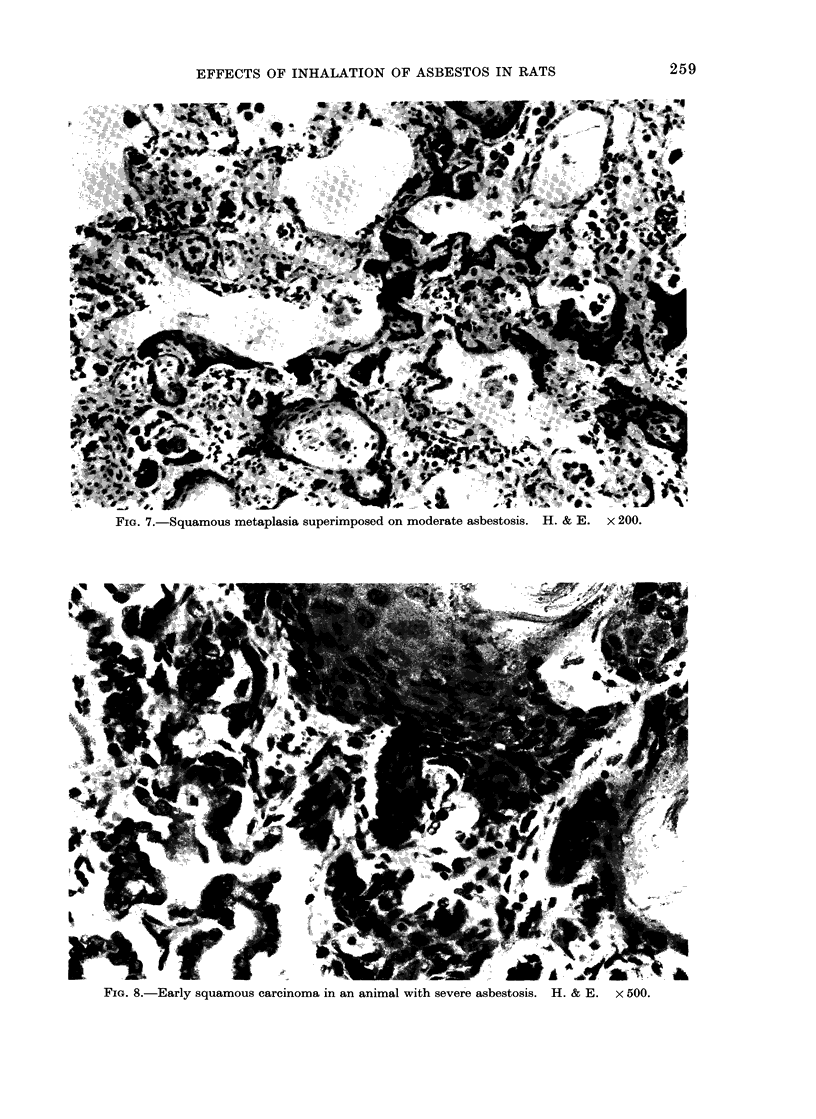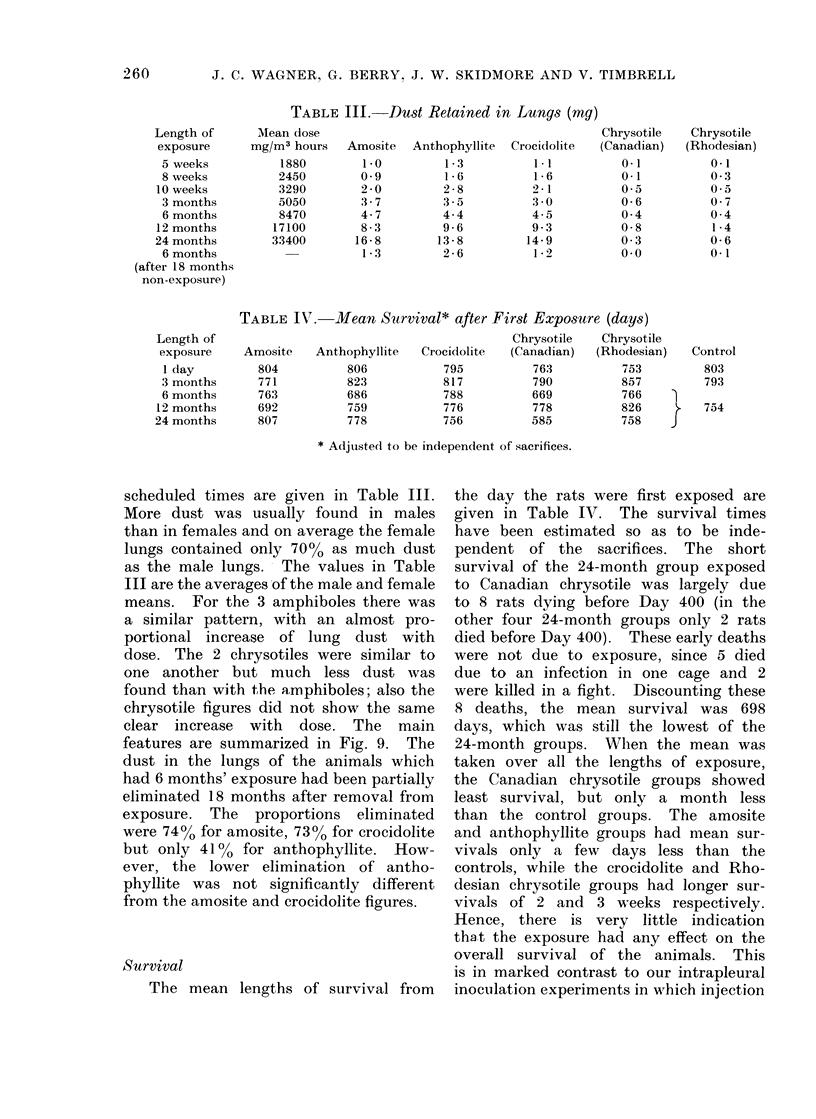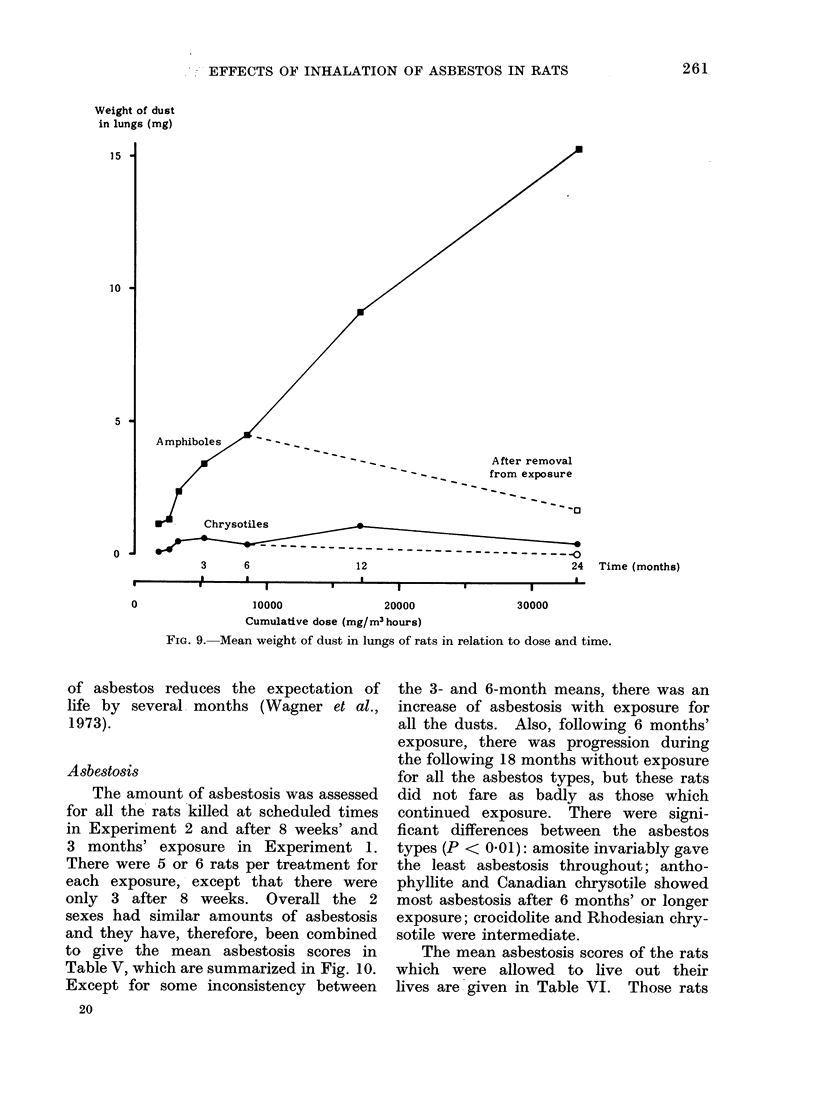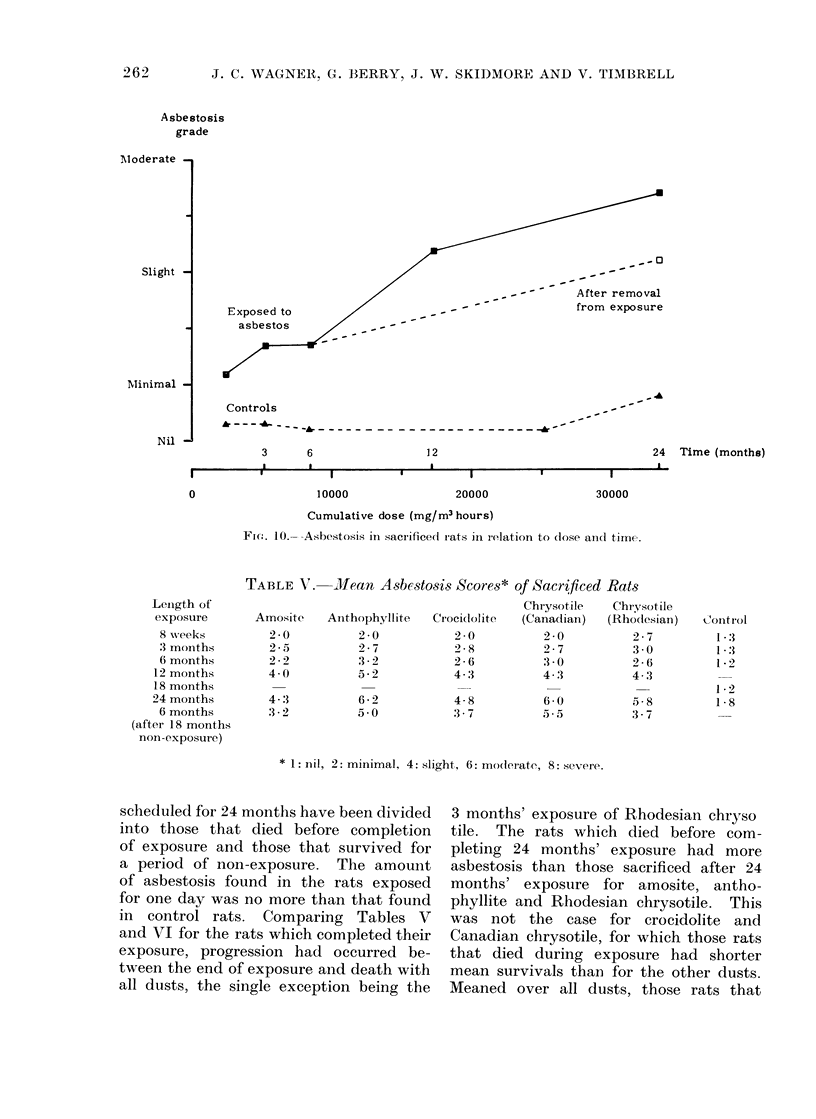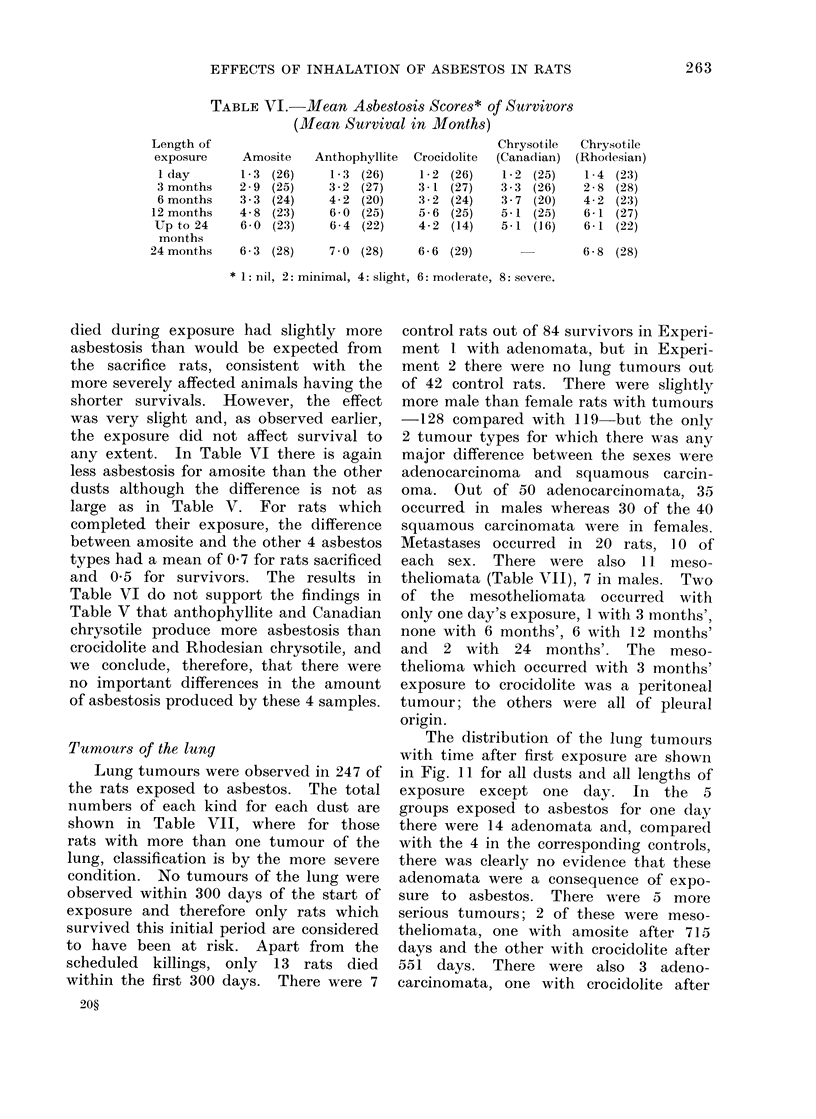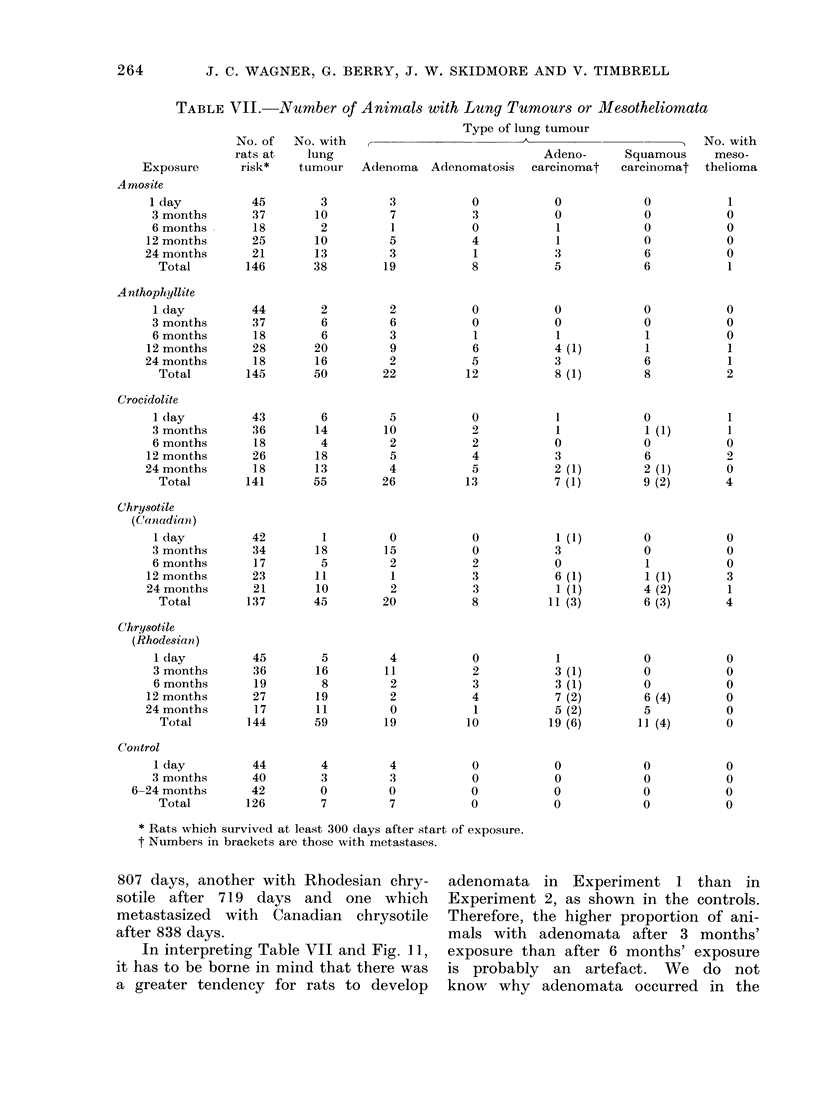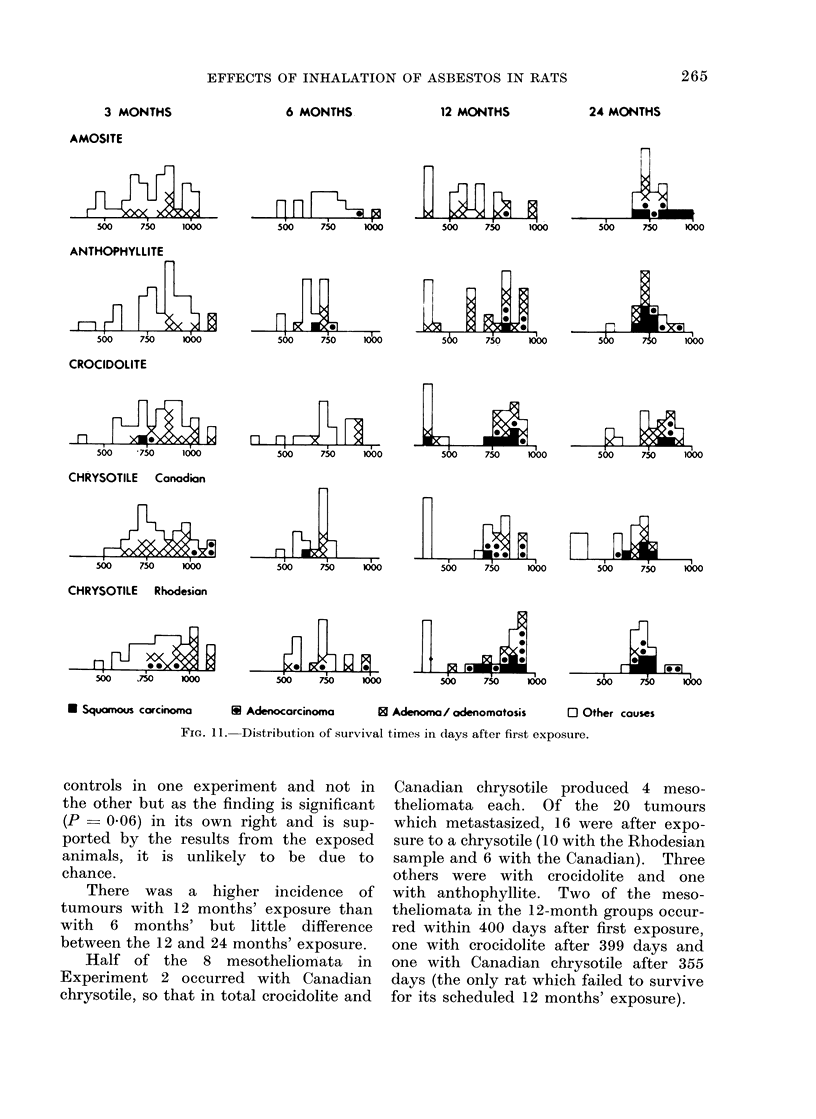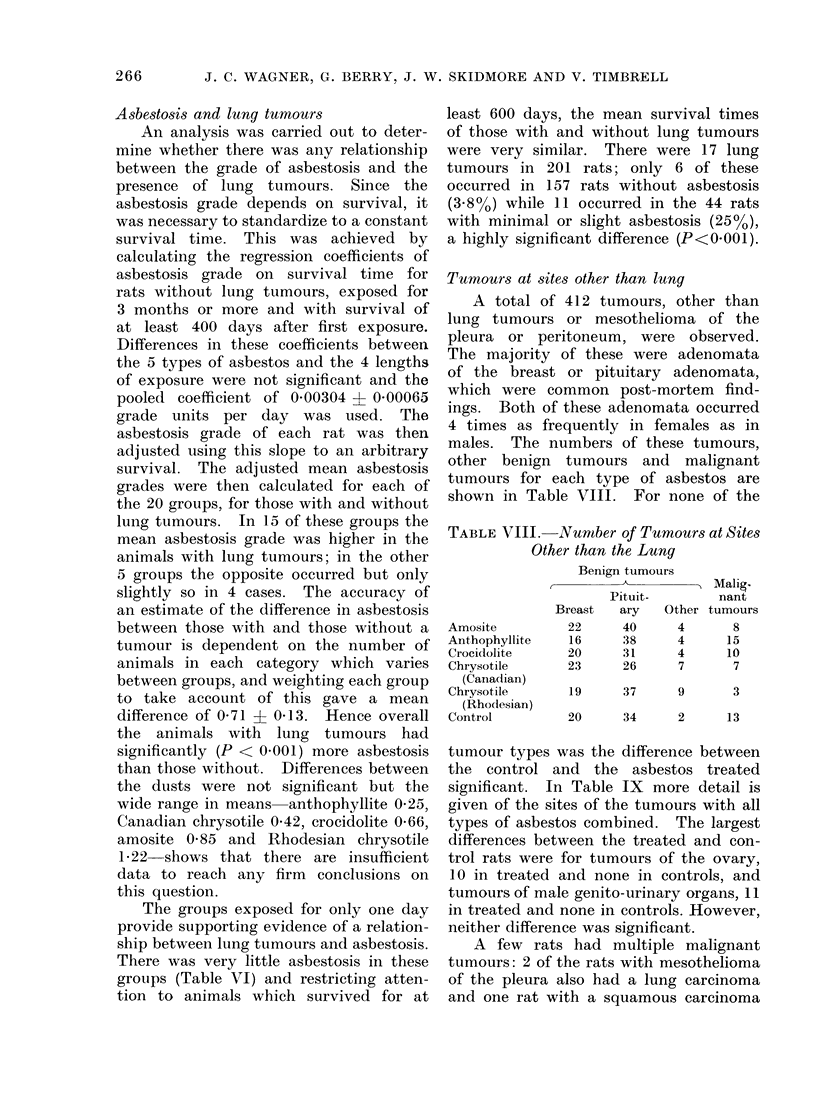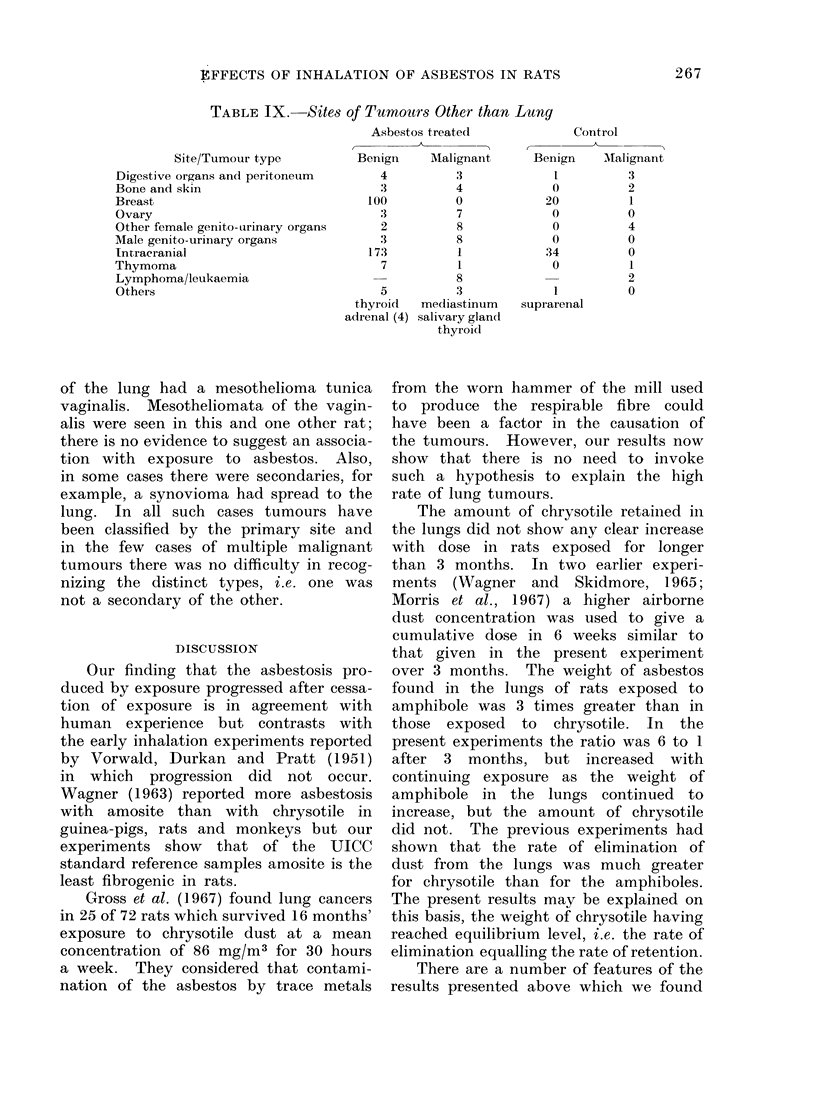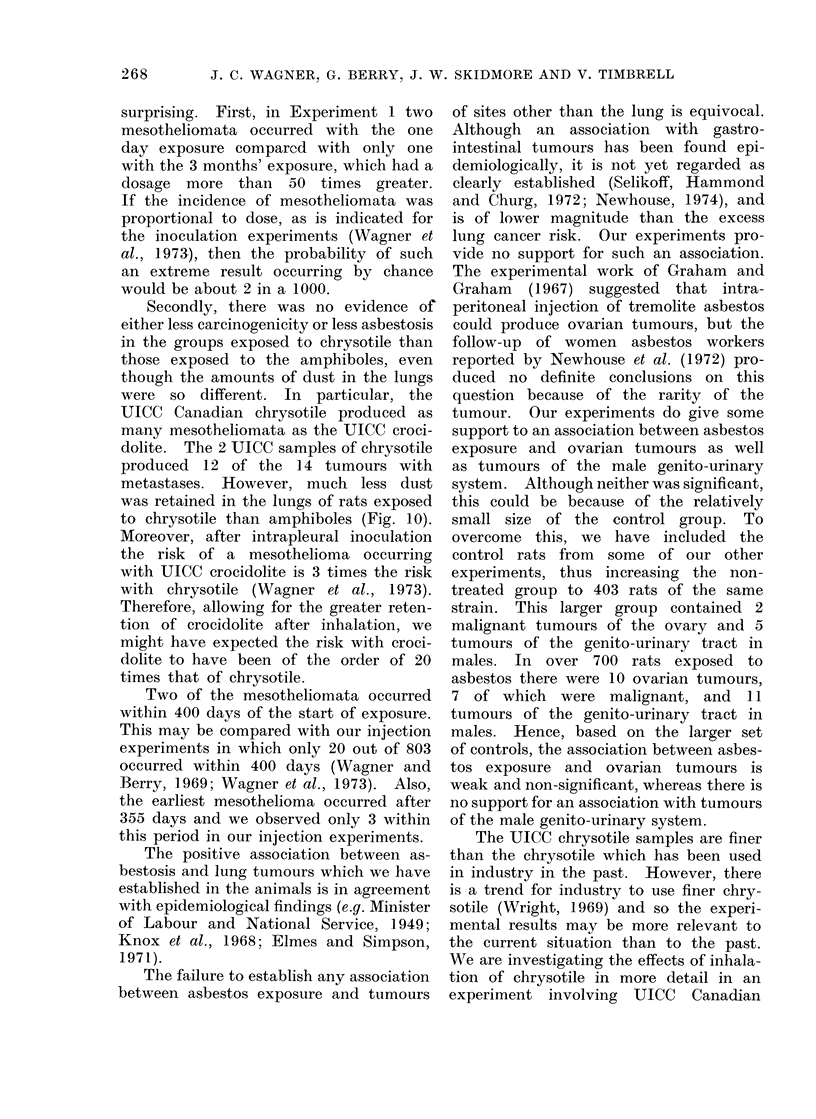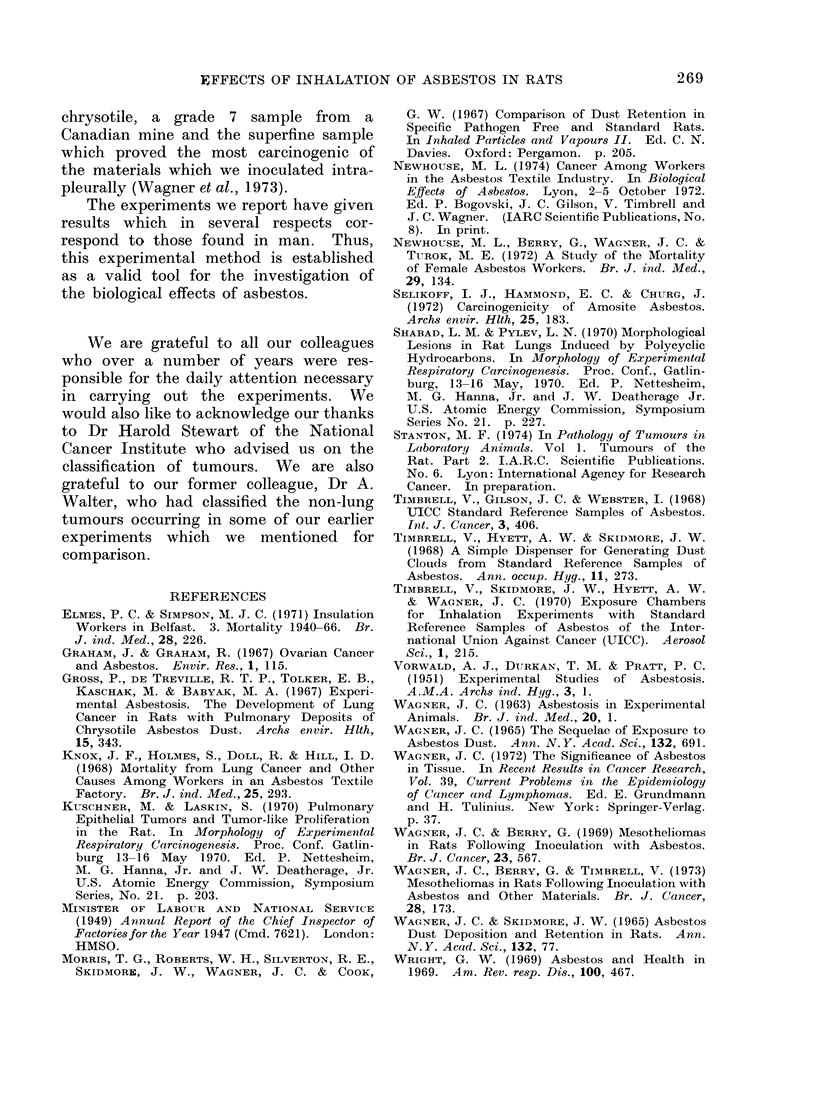# The Effects of the Inhalation of Asbestos in Rats

**DOI:** 10.1038/bjc.1974.65

**Published:** 1974-03

**Authors:** J. C. Wagner, G. Berry, J. W. Skidmore, V. Timbrell

## Abstract

**Images:**


*Br. J. Cancer* (1974) **29**, 252

### THE EFFECTS OF THE INHALATION OF ASBESTOS IN RATS

J. C. WAGNER, G. BERRY, J. W. SKIDMORE AND V. TIMBRELL

*From the Medical Research Council's Pneumoconiosis Unit, Llandough Hospital, Penarth, Glamorgan*

Received 5 September 1973. Accepted 29 November 1973

**Summary.—Two experiments in which SPF Wistar rats were exposed by inhalation to dust clouds of the UICC standard reference samples for periods of between one day and 2 years are described. All the samples of asbestos produced asbestosis which continued to progress after removal from exposure but only a little fibrosis was observed in control rats. Lung tumours, ranging in severity from adenomata to squamous carcinomata, were produced by all samples but in the controls there were only a few adenomata and none of the more serious tumours. Of the 20 tumours which metastasized, 16 occurred after exposure to one or other of the 2 chrysotile samples. In addition, a total of 11 mesotheliomata occurred, 4 of which were with crocidolite and 4 with Canadian chrysotile. Two of the mesotheliomata occurred with only one day's exposure to asbestos. There was a positive association between asbestosis and lung tumours.**

WAGNER, Berry and Timbrell (1973) reported the results of experiments in which rats were inoculated intrapleurally with samples of asbestos. In the discussion it was mentioned that two experiments in which rats had been exposed to dust clouds of the UICC reference samples had been carried out, and the results of these are now presented. Some preliminary results of one of these experiments were reported by Wagner (1972).

In both experiments the rats were exposed to similar dust concentrations and the dose varied by exposing rats for various lengths of time. The main aim was to establish the relationship between the development of malignant tumours in the lungs and the dose and type of asbestos dust but, additionally, the amount of fibrosis was assessed.

### MATERIALS AND METHODS

Caesarean derived rats of the Wistar strain were used which had been bred at the Unit from SPF stocks given to us by the Imperial Chemical Industries, Pharmaceutical Division at Alderley Edge, Cheshire in 1964 and 1968.

The asbestos samples used consisted of the 5 UICC standard reference samples (Timbrell, Gilson and Webster, 1968) which were prepared following recommendations of the UICC. These samples are of 3 amphibole types—amosite, anthophyllite and crocidolite—and 2 chrysotiles—a Canadian and a Rhodesian sample.

The rats were exposed in 1·4 $m^3$ inhalation chambers (Timbrell *et al.*, 1970) which contained 8 cages, each of which could hold 6 rats, or, for a short period with young rats, there was room for a seventh. Five chambers were used, one for each type of asbestos. The chambers were constructed so that the rats could be tended without the chambers being opened.

The dust clouds were generated using a specially devised dispenser (Timbrell, Hyett and Skidmore, 1968). The clouds were generated for 7 hours a day and 5 days per week. The respirable dust concentrations were measured using size selective gravimetric dust samples (Cassella Type 114A). The collected samples were evaluated at the end of each daily session. In order to achieve the required dosage, calculated as the product of concentration and time, variations occurring in the concentrations were corrected on the following days. At the end of exposure the rats were left in the chambers for a few days, to allow time for their fur to become clear of asbestos before transferring them to a clean environment for the remainder of their lives. At transfer some rats, generally 2 or 3 of each sex, were killed and the lungs removed for histological examination and determination of dust content.

As each type of asbestos has a considerable silicate content (about 50%) the amount of dust in the lungs was determined by first assessing its silica content and referring this value to that of the respirable dust to which the rat had been exposed. This method was used in previous experiments (Morris *et al.*, 1967).

After exposure the rats were caged in threes or fours isolated in a special unit supplied with filtered air. The inhalation chambers were also in this unit but in a separate room. They were fed on a proprietary brand of autoclaved cubes, and water *ad libitum*. Except for the scheduled killings, each rat was allowed to live until it died or appeared to be distressed, and a full necropsy examination was carried out.

*Histological preparation, staining and microscopic methods*.—For the scheduled killings, animals were killed by chloroform anaesthesia and following exsanguination the thorax opened and the lungs removed. The left lung was air inflated and suspended in formalin. Representative portions of the right lung were taken for electron microscopic examination and the remainder of the lung dilated with neutral buffered formalin. Slices were taken from both lungs for histological examination. After the histological sections had been cut, the embedded tissue and the trimmings were dewaxed and added to the remainder of the lungs which were used for the chemical estimates.

For other animals, at post mortem the lungs were dilated with neutral buffered formalin and after fixation were sliced sagittally, routine sections being taken of the whole left lung and the upper and lower lobes of the right lung. In addition, any other suspicious lesions from the lungs or other organs were taken for histological examination.

In all cases, sections were stained with haematoxylin and eosin and the lung sections were stained for elastin, reticulin and collagen. Special stains were used in some cases as an aid to diagnosis of the tumours.

*Assessment of the severity of asbestosis*.—Sections of both lungs were examined without knowledge of the duration or type of asbestos exposure and with the animals in random order. The sections were observed on a viewing screen of a Projectina Microscope 4013 BK using a ×7 objective. At this magnification a large proportion of the lung could be assessed in a single field and, as it was not possible to observe asbestos fibres at this magnification, the results were not biased by knowing the type of asbestos to which the animal had been exposed.

*Experiments*

In both experiments and for all doses there were groups which were exposed to all 5 reference samples; there were also control groups which were not exposed. Rats were allocated to treatments at random. At the start of the exposure the majority of the rats were between 5 and 7 weeks old, a few being slightly older or younger, and there were approximately equal numbers of males and females.

*Experiment* 1.—There were 2 time intervals of exposure: in the first, rats were exposed for 3 months starting in May 1967 and in the second, groups of rats were exposed for one day only in August 1967. In addition to the killings at the end of exposure, in the 3-month group there were also intermediate sacrifices at 5, 8 and 10 weeks.

*Experiment* 2.—There were 3 time intervals of exposure—6 months, 12 months and 24 months. The experiment started in January 1969 and after 6 months half of the rats were removed from the cabinets. They were replaced and a year later these replacement animals were in turn removed. They were replaced by animals to be used for special electron microscopy examinations which will not be reported in this paper. The remaining animals were removed in January 1971 after 2 years' exposure. In the 6-month groups, in addition to the killings at the end of exposure, rats were also killed after 2 years, *i.e.* 18 months after removal from exposure.

The numbers of rats are given in Table I. In the 24-month groups, overcrowding amongst the males tended to occur due to increase in size after about a year, and some were removed prematurely after 13½ months. For analysis these rats have been

TABLE I.—*Number of Rats Exposed*

| Length of exposure | Amosite | Anthophyllite | Crocidolite | Chrysotile (Canadian) | Chrysotile (Rhodesian) | Control |
|---|---|---|---|---|---|---|
| 1 day | 49 | 49 | 49 | 49 | 49 | 48 |
| 3 months | 52 | 52 | 52 | 52 | 52 | 58 |
| 6 months | 24 | 24 | 24 | 24 | 25 | |
| 12 months | 25 | 28 | 26 | 23 | 27 | 48 |
| 24 months | 21 | 19 | 20 | 24 | 20 | |

TABLE II.—*Mean Respirable Dust Concentration* (*mg*/*m*$^3$) *and Cumulative Dose* (*mg*/*m*$^3$ *hours*)

| Length of exposure | Amosite | | Anthophyllite | | Crocidolite | | Chrysotile (Canadian) | | Chrysotile (Rhodesian) | |
|---|---|---|---|---|---|---|---|---|---|---|
| 1 day | 14·1 | 99 | 12·8 | 90 | 12·5 | 88 | 9·7 | 68 | 14·7 | 103 |
| 3 months | 12·4 | 5000 | 13·5 | 5240 | 12·6 | 5030 | 12·1 | 4930 | 12·3 | 5070 |
| 6 months | 11·2 | 8550 | 10·9 | 8540 | 10·7 | 8430 | 10·2 | 8240 | 10·7 | 8590 |
| 12 months | 10·8 | 17000 | 11·4 | 17100 | 10·6 | 17000 | 10·7 | 17100 | 10·9 | 17100 |
| 24 months | 10·6 | 33500 | 10·6 | 33700 | 10·3 | 33200 | 10·1 | 33200 | 10·1 | 33600 |

included in the 12-month groups and hence the numbe1 of rats in these groups is slightly higher, and in the 24-month groups slightly lower, than planned.

In Table II the mean respirable dust concentrations and the cumulative doses, the products of concentration and time, are shown. The one-day exposure was 7 hours for all dusts. For the other 4 time intervals of exposure, there were slight variations in the number of hours required to achieve approximately equal doses but the mean times were 402, 788, 1574 and 3237 hours respectively. The mean concentrations were usually higher in Experiment 1 than Experiment 2, and the 3-month group had an average dose of 60% of that of the 6-month group. Reasonable equality of dose between the dusts was achieved for all the lengths of exposure, except for the one-day which was too short to allow any adjustments.

*Interpretation of histological findings*

*Classification of asbestosis*.—The lesions seen in the lungs of rats exposed to all types of asbestos were similar to those described in guinea-pigs (Wagner, 1963, 1965). There were 2 main differences; firstly, asbestos bodies were never seen in the lung tissue of the rat although they are frequently seen in the pleural granulomata which follow the intra-pleural inoculation of amphibole fibres; secondly, there was a far greater production of granular pneumocytes (type II) alveolar epithelial cells in the rat.

The lesions consist initially of a deposition of asbestos fibres, alveolar macrophages and cell debris in the alveoli arising directly from the respiratory bronchioles. These deposits become organized firstly by being enmeshed in a thin reticulin network which coarsens with time and becomes replaced by collagen fibres. The alveolar epithelium reacts with replacement of the type I cells, and becomes completely lined by granular pneumocytes. In some of the alveoli the epithelium is shed into the lumina, in others there is a walling of the alveoli by these cells. This usually occurs at the bifurcations where groups of alveoli are closed off from the lumina of the respiratory bronchioles, giving the so-called pseudo-acinar appearance. In the guinea-pigs these small cystic spaces contained asbestos fibre and degenerating macrophages, but in the rats numerous granular pneumocytes were also present. The initial lesions were confined to occasional discrete individual respiratory bronchioles scattered throughout the lung substance. After further exposure, more and more respiratory bronchioles become involved and all the respiratory bronchioles arising from terminal bronchioles become thickened as the fibrous tissue network extends into the wall of the respiratory bronchiole, and this interstitial reaction spreads down into the peripheral elements of the primary unit, involving the alveolar ducts, atria and finally the air sacs and alveoli. With progression, the individual lesions tend to coalesce, leading to the development of a

FIG. 1.—Slight asbestosis (Grade 4). Thickening of the walls of the alveoli arising directly from the respiratory bronchioles, with replacement of normal epithelium by type II cells. H. & E. ×80.

FIG. 2.—Slight asbestosis (Grade 4). Higher power showing numerous refractile crocidolite fibres in the alveoli of a respiratory bronchiole. Asbestos bodies are rarely seen in the lungs of rats. Illumination reduced to illustrate the fibres. H. & E. ×500.

FIG. 3.—Moderate asbestosis (Grade 6). A lower power projection illustrating that the lesion although still mainly involving the respiratory bronchiole is now diffuse. Aggregations of type II pneumocytes are seen in the lumina as well as investing the walls. H. & E. ×80.

FIG. 4.—Severe asbestosis (Grade 8). There is a generalized interstitial fibrosis. H. & E. ×80.

diffuse interstitial fibrosis with gradual increase in the density of the fibrous tissue, ultimately resulting in the replacement of most of the lung parenchyma by a dense collagen network surrounding distorted air spaces, many of which contain large clumps of granular pneumocytes which, in some areas, have lysed leaving foci of lipo-alveolar proteinosis.

The assessment of these lesions was based on 4 grades of fibrosis—minimal, slight, moderate and severe. Typical examples of slight, moderate and severe asbestosis are shown in Fig. 1–4. In addition, when carrying out the assessment it was found convenient to introduce the intermediate categories minimal/slight, slight/moderate and moderate/severe. The 7 resulting categories were scored 2–8 and the normal lung, in which there was no sign of asbestosis, was scored 1, and the assessments were averaged for each group of rats. The repeatability of this form of assessment was tested on 154 sections, reassessed in a different random order and 90% were assigned to within one category of the first reading.

*Classification of tumours.*—The tumours found in the lungs of rats after exposure to the various types of asbestos dusts were peripheral adenomata, widespread adenomatosis, adenocarcinomata and squamous carcinomata. The rarity of pulmonary tumours in rats has been stressed in the reviews by Kuschner and Laskin (1970) and Shabad and Pylev (1970). Further, these authors have described the development and morphological features of the tumours that we are reporting. All these tumours were peripheral and appeared to arise from the region of the respiratory bronchioles in which the asbestos fibre had accumulated.

The origin of the adenomata appeared to be from accumulation of type II epithelial cells that proliferated in the alveoli of the respiratory bronchioles (Fig. 5, 6). In many of the exposed animals these tumours were multiple, and in a number of animals, particularly those with the more severe grades of asbestosis, there seemed to be adenomata arising from numerous adjoining respiratory bronchioles, giving an impression of contiguous adenomata invading large areas of the lung. Dr Harold Stewart (personal communication) suggested that this type of lesion should be referred to as adenomatosis. In contrast to this, a few control animals were seen to have solitary adenomata which were small in size. The adenocarcinomata were of the same type and origin as the "alveolar adenocarcinomata" described by Shabad and Pylev (1970); many of these tumours were papillary adenoma–carcinomata. The squamous carcinomata appeared to originate from foci of squamous metaplasia occurring in the asbestotic lesions in the respiratory bronchioles (Fig. 7, 8). As far as can be ascertained, all these tumours were peripheral in origin and not bronchial papillomata. The classification of these tumours was discussed in some detail in Session VI at the Gatlinburg Conference on the Morphology of Experimental Respiratory Carcinogenesis in 1970; and the chapter by M. F. Stanton (1974) in the IARC Monograph on *Pathology of Tumours in Laboratory Animals* contains detailed descriptions of the tumours that we have illustrated.

The rats used in this experiment are from a caesarean derived, barrier maintained colony and fortunately they have been kept free of rat bronchitis; therefore the squamous metaplasia was not associated with bronchiectasis.

Metastases in the thoracic cavity to the chest wall, diaphragm, pericardium or the tracheo-bronchial lymph glands were seen in 14 animals; the majority had lesions invading 3 of the sites and in only 2 animals were metastases observed in the tracheobronchial glands. In one animal secondary deposits were seen in sections from a kidney. Eight adenocarcinomata and 6 squamous tumours had metastasized.

### RESULTS

All except 2 of the rats in the groups with exposure of 12 months or less survived for the whole of their planned exposure. In the 24-month group there was appreciable mortality before the end of exposure and only 53% survived for the full period. Out of 1013 rats it was impossible to obtain adequate histological material in only 8 because of cannibalism.

### *Dust retention*

The mean weights of asbestos dust in the lungs of animals killed at the

FIG. 5.—Papillary adenoma. H. & E. ×320.

FIG. 6.—Electron-micrograph of an adenoma showing type II pneumocytes.

FIG. 7.—Squamous metaplasia superimposed on moderate asbestosis. H. & E. ×200.

FIG. 8.—Early squamous carcinoma in an animal with severe asbestosis. H. & E. ×500.

TABLE III.—*Dust Retained in Lungs (mg)*

| Length of exposure | Mean dose mg/m³ hours | Amosite | Anthophyllite | Crocidolite | Chrysotile (Canadian) | Chrysotile (Rhodesian) |
|---|---|---|---|---|---|---|
| 5 weeks | 1880 | 1·0 | 1·3 | 1·1 | 0·1 | 0·1 |
| 8 weeks | 2450 | 0·9 | 1·6 | 1·6 | 0·1 | 0·3 |
| 10 weeks | 3290 | 2·0 | 2·8 | 2·1 | 0·5 | 0·5 |
| 3 months | 5050 | 3·7 | 3·5 | 3·0 | 0·6 | 0·7 |
| 6 months | 8470 | 4·7 | 4·4 | 4·5 | 0·4 | 0·4 |
| 12 months | 17100 | 8·3 | 9·6 | 9·3 | 0·8 | 1·4 |
| 24 months | 33400 | 16·8 | 13·8 | 14·9 | 0·3 | 0·6 |
| 6 months (after 18 months non-exposure) | — | 1·3 | 2·6 | 1·2 | 0·0 | 0·1 |

TABLE IV.—*Mean Survival* after First Exposure (days)*

| Length of exposure | Amosite | Anthophyllite | Crocidolite | Chrysotile (Canadian) | Chrysotile (Rhodesian) | Control |
|---|---|---|---|---|---|---|
| 1 day | 804 | 806 | 795 | 763 | 753 | 803 |
| 3 months | 771 | 823 | 817 | 790 | 857 | 793 |
| 6 months | 763 | 686 | 788 | 669 | 766 | 754 (6, 12 and 24 months) |
| 12 months | 692 | 759 | 776 | 778 | 826 | |
| 24 months | 807 | 778 | 756 | 585 | 758 | |

* Adjusted to be independent of sacrifices.

scheduled times are given in Table III. More dust was usually found in males than in females and on average the female lungs contained only 70% as much dust as the male lungs. The values in Table III are the averages of the male and female means. For the 3 amphiboles there was a similar pattern, with an almost proportional increase of lung dust with dose. The 2 chrysotiles were similar to one another but much less dust was found than with the amphiboles; also the chrysotile figures did not show the same clear increase with dose. The main features are summarized in Fig. 9. The dust in the lungs of the animals which had 6 months' exposure had been partially eliminated 18 months after removal from exposure. The proportions eliminated were 74% for amosite, 73% for crocidolite but only 41% for anthophyllite. However, the lower elimination of anthophyllite was not significantly different from the amosite and crocidolite figures.

*Survival*

The mean lengths of survival from the day the rats were first exposed are given in Table IV. The survival times have been estimated so as to be independent of the sacrifices. The short survival of the 24-month group exposed to Canadian chrysotile was largely due to 8 rats dying before Day 400 (in the other four 24-month groups only 2 rats died before Day 400). These early deaths were not due to exposure, since 5 died due to an infection in one cage and 2 were killed in a fight. Discounting these 8 deaths, the mean survival was 698 days, which was still the lowest of the 24-month groups. When the mean was taken over all the lengths of exposure, the Canadian chrysotile groups showed least survival, but only a month less than the control groups. The amosite and anthophyllite groups had mean survivals only a few days less than the controls, while the crocidolite and Rhodesian chrysotile groups had longer survivals of 2 and 3 weeks respectively. Hence, there is very little indication that the exposure had any effect on the overall survival of the animals. This is in marked contrast to our intrapleural inoculation experiments in which injection

FIG. 9.—Mean weight of dust in lungs of rats in relation to dose and time.

of asbestos reduces the expectation of life by several months (Wagner *et al.*, 1973).

*Asbestosis*

The amount of asbestosis was assessed for all the rats killed at scheduled times in Experiment 2 and after 8 weeks' and 3 months' exposure in Experiment 1. There were 5 or 6 rats per treatment for each exposure, except that there were only 3 after 8 weeks. Overall the 2 sexes had similar amounts of asbestosis and they have, therefore, been combined to give the mean asbestosis scores in Table V, which are summarized in Fig. 10. Except for some inconsistency between the 3- and 6-month means, there was an increase of asbestosis with exposure for all the dusts. Also, following 6 months' exposure, there was progression during the following 18 months without exposure for all the asbestos types, but these rats did not fare as badly as those which continued exposure. There were significant differences between the asbestos types ($P < 0{\cdot}01$): amosite invariably gave the least asbestosis throughout; anthophyllite and Canadian chrysotile showed most asbestosis after 6 months' or longer exposure; crocidolite and Rhodesian chrysotile were intermediate.

The mean asbestosis scores of the rats which were allowed to live out their lives are given in Table VI. Those rats

FIG. 10.—Asbestosis in sacrificed rats in relation to dose and time.

TABLE V.—*Mean Asbestosis Scores* of Sacrificed Rats*

| Length of exposure | Amosite | Anthophyllite | Crocidolite | Chrysotile (Canadian) | Chrysotile (Rhodesian) | Control |
|---|---|---|---|---|---|---|
| 8 weeks | 2·0 | 2·0 | 2·0 | 2·0 | 2·7 | 1·3 |
| 3 months | 2·5 | 2·7 | 2·8 | 2·7 | 3·0 | 1·3 |
| 6 months | 2·2 | 3·2 | 2·6 | 3·0 | 2·6 | 1·2 |
| 12 months | 4·0 | 5·2 | 4·3 | 4·3 | 4·3 | — |
| 18 months | — | — | — | — | — | 1·2 |
| 24 months | 4·3 | 6·2 | 4·8 | 6·0 | 5·8 | 1·8 |
| 6 months (after 18 months non-exposure) | 3·2 | 5·0 | 3·7 | 5·5 | 3·7 | — |

* 1: nil, 2: minimal, 4: slight, 6: moderate, 8: severe.

scheduled for 24 months have been divided into those that died before completion of exposure and those that survived for a period of non-exposure. The amount of asbestosis found in the rats exposed for one day was no more than that found in control rats. Comparing Tables V and VI for the rats which completed their exposure, progression had occurred between the end of exposure and death with all dusts, the single exception being the 3 months' exposure of Rhodesian chrysotile. The rats which died before completing 24 months' exposure had more asbestosis than those sacrificed after 24 months' exposure for amosite, anthophyllite and Rhodesian chrysotile. This was not the case for crocidolite and Canadian chrysotile, for which those rats that died during exposure had shorter mean survivals than for the other dusts. Meaned over all dusts, those rats that

TABLE VI.—*Mean Asbestosis Scores* of Survivors (Mean Survival in Months)*

| Length of exposure | Amosite | Anthophyllite | Crocidolite | Chrysotile (Canadian) | Chrysotile (Rhodesian) |
|---|---|---|---|---|---|
| 1 day | 1·3 (26) | 1·3 (26) | 1·2 (26) | 1·2 (25) | 1·4 (23) |
| 3 months | 2·9 (25) | 3·2 (27) | 3·1 (27) | 3·3 (26) | 2·8 (28) |
| 6 months | 3·3 (24) | 4·2 (20) | 3·2 (24) | 3·7 (20) | 4·2 (23) |
| 12 months | 4·8 (23) | 6·0 (25) | 5·6 (25) | 5·1 (25) | 6·1 (27) |
| Up to 24 months | 6·0 (23) | 6·4 (22) | 4·2 (14) | 5·1 (16) | 6·1 (22) |
| 24 months | 6·3 (28) | 7·0 (28) | 6·6 (29) | — | 6·8 (28) |

* 1: nil, 2: minimal, 4: slight, 6: moderate, 8: severe.

died during exposure had slightly more asbestosis than would be expected from the sacrifice rats, consistent with the more severely affected animals having the shorter survivals. However, the effect was very slight and, as observed earlier, the exposure did not affect survival to any extent. In Table VI there is again less asbestosis for amosite than the other dusts although the difference is not as large as in Table V. For rats which completed their exposure, the difference between amosite and the other 4 asbestos types had a mean of 0·7 for rats sacrificed and 0·5 for survivors. The results in Table VI do not support the findings in Table V that anthophyllite and Canadian chrysotile produce more asbestosis than crocidolite and Rhodesian chrysotile, and we conclude, therefore, that there were no important differences in the amount of asbestosis produced by these 4 samples.

### *Tumours of the lung*

Lung tumours were observed in 247 of the rats exposed to asbestos. The total numbers of each kind for each dust are shown in Table VII, where for those rats with more than one tumour of the lung, classification is by the more severe condition. No tumours of the lung were observed within 300 days of the start of exposure and therefore only rats which survived this initial period are considered to have been at risk. Apart from the scheduled killings, only 13 rats died within the first 300 days. There were 7 control rats out of 84 survivors in Experiment 1 with adenomata, but in Experiment 2 there were no lung tumours out of 42 control rats. There were slightly more male than female rats with tumours —128 compared with 119—but the only 2 tumour types for which there was any major difference between the sexes were adenocarcinoma and squamous carcinoma. Out of 50 adenocarcinomata, 35 occurred in males whereas 30 of the 40 squamous carcinomata were in females. Metastases occurred in 20 rats, 10 of each sex. There were also 11 mesotheliomata (Table VII), 7 in males. Two of the mesotheliomata occurred with only one day's exposure, 1 with 3 months', none with 6 months', 6 with 12 months' and 2 with 24 months'. The mesothelioma which occurred with 3 months' exposure to crocidolite was a peritoneal tumour; the others were all of pleural origin.

The distribution of the lung tumours with time after first exposure are shown in Fig. 11 for all dusts and all lengths of exposure except one day. In the 5 groups exposed to asbestos for one day there were 14 adenomata and, compared with the 4 in the corresponding controls, there was clearly no evidence that these adenomata were a consequence of exposure to asbestos. There were 5 more serious tumours; 2 of these were mesotheliomata, one with amosite after 715 days and the other with crocidolite after 551 days. There were also 3 adenocarcinomata, one with crocidolite after

TABLE VII.—*Number of Animals with Lung Tumours or Mesotheliomata*

| Exposure | No. of rats at risk* | No. with lung tumour | Type of lung tumour: Adenoma | Adenomatosis | Adeno-carcinoma† | Squamous carcinoma† | No. with meso-thelioma |
|---|---|---|---|---|---|---|---|
| *Amosite* | | | | | | | |
| 1 day | 45 | 3 | 3 | 0 | 0 | 0 | 1 |
| 3 months | 37 | 10 | 7 | 3 | 0 | 0 | 0 |
| 6 months | 18 | 2 | 1 | 0 | 1 | 0 | 0 |
| 12 months | 25 | 10 | 5 | 4 | 1 | 0 | 0 |
| 24 months | 21 | 13 | 3 | 1 | 3 | 6 | 0 |
| Total | 146 | 38 | 19 | 8 | 5 | 6 | 1 |
| *Anthophyllite* | | | | | | | |
| 1 day | 44 | 2 | 2 | 0 | 0 | 0 | 0 |
| 3 months | 37 | 6 | 6 | 0 | 0 | 0 | 0 |
| 6 months | 18 | 6 | 3 | 1 | 1 | 1 | 0 |
| 12 months | 28 | 20 | 9 | 6 | 4 (1) | 1 | 1 |
| 24 months | 18 | 16 | 2 | 5 | 3 | 6 | 1 |
| Total | 145 | 50 | 22 | 12 | 8 (1) | 8 | 2 |
| *Crocidolite* | | | | | | | |
| 1 day | 43 | 6 | 5 | 0 | 1 | 0 | 1 |
| 3 months | 36 | 14 | 10 | 2 | 1 | 1 (1) | 1 |
| 6 months | 18 | 4 | 2 | 2 | 0 | 0 | 0 |
| 12 months | 26 | 18 | 5 | 4 | 3 | 6 | 2 |
| 24 months | 18 | 13 | 4 | 5 | 2 (1) | 2 (1) | 0 |
| Total | 141 | 55 | 26 | 13 | 7 (1) | 9 (2) | 4 |
| *Chrysotile (Canadian)* | | | | | | | |
| 1 day | 42 | 1 | 0 | 0 | 1 (1) | 0 | 0 |
| 3 months | 34 | 18 | 15 | 0 | 3 | 0 | 0 |
| 6 months | 17 | 5 | 2 | 2 | 0 | 1 | 0 |
| 12 months | 23 | 11 | 1 | 3 | 6 (1) | 1 (1) | 3 |
| 24 months | 21 | 10 | 2 | 3 | 1 (1) | 4 (2) | 1 |
| Total | 137 | 45 | 20 | 8 | 11 (3) | 6 (3) | 4 |
| *Chrysotile (Rhodesian)* | | | | | | | |
| 1 day | 45 | 5 | 4 | 0 | 1 | 0 | 0 |
| 3 months | 36 | 16 | 11 | 2 | 3 (1) | 0 | 0 |
| 6 months | 19 | 8 | 2 | 3 | 3 (1) | 0 | 0 |
| 12 months | 27 | 19 | 2 | 4 | 7 (2) | 6 (4) | 0 |
| 24 months | 17 | 11 | 0 | 1 | 5 (2) | 5 | 0 |
| Total | 144 | 59 | 19 | 10 | 19 (6) | 11 (4) | 0 |
| *Control* | | | | | | | |
| 1 day | 44 | 4 | 4 | 0 | 0 | 0 | 0 |
| 3 months | 40 | 3 | 3 | 0 | 0 | 0 | 0 |
| 6–24 months | 42 | 0 | 0 | 0 | 0 | 0 | 0 |
| Total | 126 | 7 | 7 | 0 | 0 | 0 | 0 |

* Rats which survived at least 300 days after start of exposure.
† Numbers in brackets are those with metastases.

807 days, another with Rhodesian chrysotile after 719 days and one which metastasized with Canadian chrysotile after 838 days.

In interpreting Table VII and Fig. 11, it has to be borne in mind that there was a greater tendency for rats to develop adenomata in Experiment 1 than in Experiment 2, as shown in the controls. Therefore, the higher proportion of animals with adenomata after 3 months' exposure than after 6 months' exposure is probably an artefact. We do not know why adenomata occurred in the

FIG. 11.—Distribution of survival times in days after first exposure.

controls in one experiment and not in the other but as the finding is significant ($P = 0{\cdot}06$) in its own right and is supported by the results from the exposed animals, it is unlikely to be due to chance.

There was a higher incidence of tumours with 12 months' exposure than with 6 months' but little difference between the 12 and 24 months' exposure.

Half of the 8 mesotheliomata in Experiment 2 occurred with Canadian chrysotile, so that in total crocidolite and Canadian chrysotile produced 4 mesotheliomata each. Of the 20 tumours which metastasized, 16 were after exposure to a chrysotile (10 with the Rhodesian sample and 6 with the Canadian). Three others were with crocidolite and one with anthophyllite. Two of the mesotheliomata in the 12-month groups occurred within 400 days after first exposure, one with crocidolite after 399 days and one with Canadian chrysotile after 355 days (the only rat which failed to survive for its scheduled 12 months' exposure).

*Asbestosis and lung tumours*

An analysis was carried out to determine whether there was any relationship between the grade of asbestosis and the presence of lung tumours. Since the asbestosis grade depends on survival, it was necessary to standardize to a constant survival time. This was achieved by calculating the regression coefficients of asbestosis grade on survival time for rats without lung tumours, exposed for 3 months or more and with survival of at least 400 days after first exposure. Differences in these coefficients between the 5 types of asbestos and the 4 lengths of exposure were not significant and the pooled coefficient of $0{\cdot}00304 \pm 0{\cdot}00065$ grade units per day was used. The asbestosis grade of each rat was then adjusted using this slope to an arbitrary survival. The adjusted mean asbestosis grades were then calculated for each of the 20 groups, for those with and without lung tumours. In 15 of these groups the mean asbestosis grade was higher in the animals with lung tumours; in the other 5 groups the opposite occurred but only slightly so in 4 cases. The accuracy of an estimate of the difference in asbestosis between those with and those without a tumour is dependent on the number of animals in each category which varies between groups, and weighting each group to take account of this gave a mean difference of $0{\cdot}71 \pm 0{\cdot}13$. Hence overall the animals with lung tumours had significantly ($P < 0{\cdot}001$) more asbestosis than those without. Differences between the dusts were not significant but the wide range in means—anthophyllite 0·25, Canadian chrysotile 0·42, crocidolite 0·66, amosite 0·85 and Rhodesian chrysotile 1·22—shows that there are insufficient data to reach any firm conclusions on this question.

The groups exposed for only one day provide supporting evidence of a relationship between lung tumours and asbestosis. There was very little asbestosis in these groups (Table VI) and restricting attention to animals which survived for at least 600 days, the mean survival times of those with and without lung tumours were very similar. There were 17 lung tumours in 201 rats; only 6 of these occurred in 157 rats without asbestosis (3·8%) while 11 occurred in the 44 rats with minimal or slight asbestosis (25%), a highly significant difference ($P<0{\cdot}001$).

*Tumours at sites other than lung*

A total of 412 tumours, other than lung tumours or mesothelioma of the pleura or peritoneum, were observed. The majority of these were adenomata of the breast or pituitary adenomata, which were common post-mortem findings. Both of these adenomata occurred 4 times as frequently in females as in males. The numbers of these tumours, other benign tumours and malignant tumours for each type of asbestos are shown in Table VIII. For none of the tumour types was the difference between the control and the asbestos treated significant. In Table IX more detail is given of the sites of the tumours with all types of asbestos combined. The largest differences between the treated and control rats were for tumours of the ovary, 10 in treated and none in controls, and tumours of male genito-urinary organs, 11 in treated and none in controls. However, neither difference was significant.

TABLE VIII.—*Number of Tumours at Sites Other than the Lung*

| | Benign tumours | | | |
|---|---|---|---|---|
| | Breast | Pituitary | Other | Malignant tumours |
| Amosite | 22 | 40 | 4 | 8 |
| Anthophyllite | 16 | 38 | 4 | 15 |
| Crocidolite | 20 | 31 | 4 | 10 |
| Chrysotile (Canadian) | 23 | 26 | 7 | 7 |
| Chrysotile (Rhodesian) | 19 | 37 | 9 | 3 |
| Control | 20 | 34 | 2 | 13 |

A few rats had multiple malignant tumours: 2 of the rats with mesothelioma of the pleura also had a lung carcinoma and one rat with a squamous carcinoma

TABLE IX.—*Sites of Tumours Other than Lung*

| Site/Tumour type | Asbestos treated Benign | Asbestos treated Malignant | Control Benign | Control Malignant |
|---|---|---|---|---|
| Digestive organs and peritoneum | 4 | 3 | 1 | 3 |
| Bone and skin | 3 | 4 | 0 | 2 |
| Breast | 100 | 0 | 20 | 1 |
| Ovary | 3 | 7 | 0 | 0 |
| Other female genito-urinary organs | 2 | 8 | 0 | 4 |
| Male genito-urinary organs | 3 | 8 | 0 | 0 |
| Intracranial | 173 | 1 | 34 | 0 |
| Thymoma | 7 | 1 | 0 | 1 |
| Lymphoma/leukaemia | — | 8 | — | 2 |
| Others | 5 thyroid adrenal (4) | 3 mediastinum salivary gland thyroid | 1 suprarenal | 0 |

of the lung had a mesothelioma tunica vaginalis. Mesotheliomata of the vaginalis were seen in this and one other rat; there is no evidence to suggest an association with exposure to asbestos. Also, in some cases there were secondaries, for example, a synovioma had spread to the lung. In all such cases tumours have been classified by the primary site and in the few cases of multiple malignant tumours there was no difficulty in recognizing the distinct types, *i.e.* one was not a secondary of the other.

### DISCUSSION

Our finding that the asbestosis produced by exposure progressed after cessation of exposure is in agreement with human experience but contrasts with the early inhalation experiments reported by Vorwald, Durkan and Pratt (1951) in which progression did not occur. Wagner (1963) reported more asbestosis with amosite than with chrysotile in guinea-pigs, rats and monkeys but our experiments show that of the UICC standard reference samples amosite is the least fibrogenic in rats.

Gross *et al.* (1967) found lung cancers in 25 of 72 rats which survived 16 months' exposure to chrysotile dust at a mean concentration of 86 mg/m$^3$ for 30 hours a week. They considered that contamination of the asbestos by trace metals from the worn hammer of the mill used to produce the respirable fibre could have been a factor in the causation of the tumours. However, our results now show that there is no need to invoke such a hypothesis to explain the high rate of lung tumours.

The amount of chrysotile retained in the lungs did not show any clear increase with dose in rats exposed for longer than 3 months. In two earlier experiments (Wagner and Skidmore, 1965; Morris *et al.*, 1967) a higher airborne dust concentration was used to give a cumulative dose in 6 weeks similar to that given in the present experiment over 3 months. The weight of asbestos found in the lungs of rats exposed to amphibole was 3 times greater than in those exposed to chrysotile. In the present experiments the ratio was 6 to 1 after 3 months, but increased with continuing exposure as the weight of amphibole in the lungs continued to increase, but the amount of chrysotile did not. The previous experiments had shown that the rate of elimination of dust from the lungs was much greater for chrysotile than for the amphiboles. The present results may be explained on this basis, the weight of chrysotile having reached equilibrium level, *i.e.* the rate of elimination equalling the rate of retention.

There are a number of features of the results presented above which we found surprising. First, in Experiment 1 two mesotheliomata occurred with the one day exposure compared with only one with the 3 months' exposure, which had a dosage more than 50 times greater. If the incidence of mesotheliomata was proportional to dose, as is indicated for the inoculation experiments (Wagner *et al.*, 1973), then the probability of such an extreme result occurring by chance would be about 2 in a 1000.

Secondly, there was no evidence of either less carcinogenicity or less asbestosis in the groups exposed to chrysotile than those exposed to the amphiboles, even though the amounts of dust in the lungs were so different. In particular, the UICC Canadian chrysotile produced as many mesotheliomata as the UICC crocidolite. The 2 UICC samples of chrysotile produced 12 of the 14 tumours with metastases. However, much less dust was retained in the lungs of rats exposed to chrysotile than amphiboles (Fig. 10). Moreover, after intrapleural inoculation the risk of a mesothelioma occurring with UICC crocidolite is 3 times the risk with chrysotile (Wagner *et al.*, 1973). Therefore, allowing for the greater retention of crocidolite after inhalation, we might have expected the risk with crocidolite to have been of the order of 20 times that of chrysotile.

Two of the mesotheliomata occurred within 400 days of the start of exposure. This may be compared with our injection experiments in which only 20 out of 803 occurred within 400 days (Wagner and Berry, 1969; Wagner *et al.*, 1973). Also, the earliest mesothelioma occurred after 355 days and we observed only 3 within this period in our injection experiments.

The positive association between asbestosis and lung tumours which we have established in the animals is in agreement with epidemiological findings (*e.g.* Minister of Labour and National Service, 1949; Knox *et al.*, 1968; Elmes and Simpson, 1971).

The failure to establish any association between asbestos exposure and tumours of sites other than the lung is equivocal. Although an association with gastro-intestinal tumours has been found epidemiologically, it is not yet regarded as clearly established (Selikoff, Hammond and Churg, 1972; Newhouse, 1974), and is of lower magnitude than the excess lung cancer risk. Our experiments provide no support for such an association. The experimental work of Graham and Graham (1967) suggested that intraperitoneal injection of tremolite asbestos could produce ovarian tumours, but the follow-up of women asbestos workers reported by Newhouse *et al.* (1972) produced no definite conclusions on this question because of the rarity of the tumour. Our experiments do give some support to an association between asbestos exposure and ovarian tumours as well as tumours of the male genito-urinary system. Although neither was significant, this could be because of the relatively small size of the control group. To overcome this, we have included the control rats from some of our other experiments, thus increasing the non-treated group to 403 rats of the same strain. This larger group contained 2 malignant tumours of the ovary and 5 tumours of the genito-urinary tract in males. In over 700 rats exposed to asbestos there were 10 ovarian tumours, 7 of which were malignant, and 11 tumours of the genito-urinary tract in males. Hence, based on the larger set of controls, the association between asbestos exposure and ovarian tumours is weak and non-significant, whereas there is no support for an association with tumours of the male genito-urinary system.

The UICC chrysotile samples are finer than the chrysotile which has been used in industry in the past. However, there is a trend for industry to use finer chrysotile (Wright, 1969) and so the experimental results may be more relevant to the current situation than to the past. We are investigating the effects of inhalation of chrysotile in more detail in an experiment involving UICC Canadian chrysotile, a grade 7 sample from a Canadian mine and the superfine sample which proved the most carcinogenic of the materials which we inoculated intrapleurally (Wagner *et al.*, 1973).

The experiments we report have given results which in several respects correspond to those found in man. Thus, this experimental method is established as a valid tool for the investigation of the biological effects of asbestos.

We are grateful to all our colleagues who over a number of years were responsible for the daily attention necessary in carrying out the experiments. We would also like to acknowledge our thanks to Dr Harold Stewart of the National Cancer Institute who advised us on the classification of tumours. We are also grateful to our former colleague, Dr A. Walter, who had classified the non-lung tumours occurring in some of our earlier experiments which we mentioned for comparison.

### REFERENCES

Elmes, P. C. & Simpson, M. J. C. (1971) Insulation Workers in Belfast. 3. Mortality 1940–66. *Br. J. ind. Med.*, **28**, 226.

Graham, J. & Graham, R. (1967) Ovarian Cancer and Asbestos. *Envir. Res.*, **1**, 115.

Gross, P., de Treville, R. T. P., Tolker, E. B., Kaschak, M. & Babyak, M. A. (1967) Experimental Asbestosis. The Development of Lung Cancer in Rats with Pulmonary Deposits of Chrysotile Asbestos Dust. *Archs envir. Hlth*, **15**, 343.

Knox, J. F., Holmes, S., Doll, R. & Hill, I. D. (1968) Mortality from Lung Cancer and Other Causes Among Workers in an Asbestos Textile Factory. *Br. J. ind. Med.*, **25**, 293.

Kuschner, M. & Laskin, S. (1970) Pulmonary Epithelial Tumors and Tumor-like Proliferation in the Rat. In *Morphology of Experimental Respiratory Carcinogenesis.* Proc. Conf. Gatlinburg 13–16 May 1970. Ed. P. Nettesheim, M. G. Hanna, Jr. and J. W. Deatherage, Jr. U.S. Atomic Energy Commission, Symposium Series, No. 21. p. 203.

Minister of Labour and National Service (1949) *Annual Report of the Chief Inspector of Factories for the Year* 1947 (Cmd. 7621). London: HMSO.

Morris, T. G., Roberts, W. H., Silverton, R. E., Skidmore, J. W., Wagner, J. C. & Cook, G. W. (1967) Comparison of Dust Retention in Specific Pathogen Free and Standard Rats. In *Inhaled Particles and Vapours II.* Ed. C. N. Davies. Oxford: Pergamon. p. 205.

Newhouse, M. L. (1974) Cancer Among Workers in the Asbestos Textile Industry. In *Biological Effects of Asbestos.* Lyon, 2–5 October 1972. Ed. P. Bogovski, J. C. Gilson, V. Timbrell and J. C. Wagner. (IARC Scientific Publications, No. 8). In print.

Newhouse, M. L., Berry, G., Wagner, J. C. & Turok, M. E. (1972) A Study of the Mortality of Female Asbestos Workers. *Br. J. ind. Med.*, **29**, 134.

Selikoff, I. J., Hammond, E. C. & Churg, J. (1972) Carcinogenicity of Amosite Asbestos. *Archs envir. Hlth*, **25**, 183.

Shabad, L. M. & Pylev, L. N. (1970) Morphological Lesions in Rat Lungs Induced by Polycyclic Hydrocarbons. In *Morphology of Experimental Respiratory Carcinogenesis.* Proc. Conf., Gatlinburg, 13–16 May, 1970. Ed. P. Nettesheim, M. G. Hanna, Jr. and J. W. Deatherage Jr. U.S. Atomic Energy Commission, Symposium Series No. 21. p. 227.

Stanton, M. F. (1974) In *Pathology of Tumours in Laboratory Animals.* Vol 1. Tumours of the Rat. Part 2. I.A.R.C. Scientific Publications. No. 6. Lyon: International Agency for Research Cancer. In preparation.

Timbrell, V., Gilson, J. C. & Webster, I. (1968) UICC Standard Reference Samples of Asbestos. *Int. J. Cancer*, **3**, 406.

Timbrell, V., Hyett, A. W. & Skidmore, J. W. (1968) A Simple Dispenser for Generating Dust Clouds from Standard Reference Samples of Asbestos. *Ann. occup. Hyg.*, **11**, 273.

Timbrell, V., Skidmore, J. W., Hyett, A. W. & Wagner, J. C. (1970) Exposure Chambers for Inhalation Experiments with Standard Reference Samples of Asbestos of the International Union Against Cancer (UICC). *Aerosol Sci.*, **1**, 215.

Vorwald, A. J., Durkan, T. M. & Pratt, P. C. (1951) Experimental Studies of Asbestosis. *A.M.A. Archs ind. Hyg.*, **3**, 1.

Wagner, J. C. (1963) Asbestosis in Experimental Animals. *Br. J. ind. Med.*, **20**, 1.

Wagner, J. C. (1965) The Sequelae of Exposure to Asbestos Dust. *Ann. N.Y. Acad. Sci.*, **132**, 691.

Wagner, J. C. (1972) The Significance of Asbestos in Tissue. In *Recent Results in Cancer Research, Vol.* 39, *Current Problems in the Epidemiology of Cancer and Lymphomas.* Ed. E. Grundmann and H. Tulinius. New York: Springer-Verlag. p. 37.

Wagner, J. C. & Berry, G. (1969) Mesotheliomas in Rats Following Inoculation with Asbestos. *Br. J. Cancer*, **23**, 567.

Wagner, J. C., Berry, G. & Timbrell, V. (1973) Mesotheliomas in Rats Following Inoculation with Asbestos and Other Materials. *Br. J. Cancer*, **28**, 173.

Wagner, J. C. & Skidmore, J. W. (1965) Asbestos Dust Deposition and Retention in Rats. *Ann. N.Y. Acad. Sci.*, **132**, 77.

Wright, G. W. (1969) Asbestos and Health in 1969. *Am. Rev. resp. Dis.*, **100**, 467.